# The Endoplasmic Reticulum Cargo Receptor FgErv14 Regulates DON Production, Growth and Virulence in *Fusarium graminearum*

**DOI:** 10.3390/life12060799

**Published:** 2022-05-27

**Authors:** Fengjiang Sun, Beibei Lv, Xuemeng Zhang, Chenyu Wang, Liyuan Zhang, Xiaochen Chen, Yuancun Liang, Lei Chen, Shenshen Zou, Hansong Dong

**Affiliations:** 1 Department of Plant Pathology, College of Plant Protection, Shandong Agricultural University, Tai’an 271018, China; 2020110381@sdau.edu.cn (F.S.); 2020110385@sdau.edu.cn (X.Z.); 2019010093@sdau.edu.cn (C.W.); lyzhang@sdau.edu.cn (L.Z.); chenxc66@sdau.edu.cn (X.C.); liangyc@sdau.edu.cn (Y.L.); hsdong@sdau.edu.cn (H.D.); 2 Key Laboratory of Agricultural Genetics and Breeding, Biotechnology Research Institute, Shanghai Academy of Agricultural Sciences, Shanghai 201106, China; lvbeibei@saas.sh.cn; 3 State Key Laboratory of Crop Biology, Shandong Agricultural University, Tai’an 271018, China

**Keywords:** FgErv14, virulence, secretory pathway, ER cargo receptor, *Fusarium graminearum*

## Abstract

*Fusarium graminearum* is a plant filamentous pathogenic fungi and the predominant causal agent of *Fusarium* head blight (FHB) in cereals worldwide. The regulators of the secretory pathway contribute significantly to fungal mycotoxin synthesis, development, and virulence. However, their roles in these processes in *F. graminearum* remain poorly understood. Here, we identified and functionally characterized the endoplasmic reticulum (ER) cargo receptor FgErv14 in *F. graminearum*. Firstly, it was observed that FgErv14 is mainly localized in the ER. Then, we constructed the FgErv14 deletion mutant (Δ*Fgerv14*) and found that the absence of the FgErv14 caused a serious reduction in vegetative growth, significant defects in asexual and sexual reproduction, and severely impaired virulence. Furthermore, we found that the Δ*Fgerv14* mutant exhibited a reduced expression of *TRI* genes and defective toxisome generation, both of which are critical for deoxynivalenol (DON) biosynthesis. Importantly, we found the green fluorescent protein (GFP)-tagged FgRud3 was dispersed in the cytoplasm, whereas GFP-FgSnc1-PEM was partially trapped in the late Golgi in Δ*Fgerv14* mutant. These results demonstrate that FgErv14 mediates anterograde ER-to-Golgi transport as well as late secretory Golgi-to-Plasma membrane transport and is necessary for DON biosynthesis, asexual and sexual reproduction, vegetative growth, and pathogenicity in *F. graminearum*.

## 1. Introduction

*Fusarium graminearum* (teleomorph: *Gibberella zeae*) is a devastating fungal pathogen that causes *Fusarium* head blight (FHB) in cereal crops, including wheat, barley, and maize [1,2,3]. FHB has been spreading worldwide, leading to severe yield losses [4,5]. In addition, *F. graminearum* produces a kind of trichothecene mycotoxin named deoxynivalenol (DON), which is toxic to humans and livestock by binding to the ribosome to inhibit the protein synthesis of eukaryotic organisms [6,7]. However, the effective control of FHB remains challenging, with management relying mainly on fungicides, which have many unwanted effects [8,9]. Therefore, understanding the underlying mechanisms responsible for the virulence in *F. graminearum* may facilitate the development of novel strategies and effective means for FHB control.

*F. graminearum* infects flowering wheat spikelets and grows through the rachis node at the bottom of each spikelet so as to colonize the entire wheat head [10]. During plant infection, *F. graminearum* produces DON, a secondary metabolite that acts as an important virulence factor to facilitate the expansion of the pathogen in wheat heads [11]. Moreover, DON seriously contaminates cereals and cereal products [12]. Traditional physical and chemical methods can remove DON to a certain extent, but these methods are not suitable for the digestion of DON from food or feed [13]. A gene, *FHB7*, which is transferred from *Thinopyrum* to wheat, encodes a Gluthione S-transferase and was found to catalyze DON into DON-GSH, thereby eliminating the toxicity of DON [14]. This discovery provides a new strategy for the safe and efficient neutralization of DON. In *F. graminearum*, DON biosynthesis is regulated by the trichothecene (*TRI*) gene cluster [15,16]. Among these genes, *TRI1* and *TRI4* encode cytochrome P-450 oxygenases localized on the toxisome that are involved in toxisome function [17,18,19,20]. *TRI5,* the first gene identified in DON biosynthesis, encodes a trichodiene synthase that is essential for the first step in the DON biosynthetic pathway [21,22]. *TRI6* and *TRI10* encode transcriptional regulators to control DON biosynthesis by regulating the *TRI* gene cluster expression [23,24]. In addition to coding products of these *TRI* genes, the proteins involved in vesicle transport have been reported as essential regulators of DON biosynthesis, which play critical roles in the pathogenesis of phytopathogenic fungi [25,26,27,28,29,30]. However, the regulatory mechanisms of these proteins are different. Many secretory proteins are synthesized in the endoplasmic reticulum (ER) and then transported out of it by a coat complex termed COPII [31]. The COPII regulators play an important role in the pathogenicity of phytopathogenic fungi. For example, in *Saccharomyces cerevisiae*, the small GTPase Rab1 has been observed to regulate the tethering of ER-derived COPII vesicles to Golgi in vitro [32,33]. In *F. graminearum*, FgRab1 is required for virulence of pathogens [34]. Another example in *F. graminearum* is the R-SNARE protein FgSec22, a subunit of the ER-to-Golgi SNARE complex, which mediates COPII-associated membrane fusion [35]. It has been reported that FgSec22 participated in the production of DON, vegetative growth, and pathogenicity in *F. graminearum* [36]. Moreover, in *Sclerotinia sclerotiorum*, SsEmp24, a protein associated with ER-derived COPII-coated vesicles, is involved in regulating pathogenicity [37]. Together, these data imply that COPII vesicles play an important role in virulence, but the roles of many COPII components are as yet unclear.

ER-derived vesicles selectively loaded with cargo interact directly or indirectly with COPII subunits [38]. Previous studies showed that in some instances, ER cargo receptors are required for linking protein cargos to coats of COPII vesicle [39,40]. In *S. cerevisiae*, ER vesicle (Erv) proteins link protein cargos to subunits of COPII vesicle coat proteins and function in transporting cargo from the ER to the Golgi [38,41,42]. The ER cargo receptor Erv14 is an integral membrane protein of *S. cerevisiae* that regulates the COPII cargo selection. Erv14 has been shown to link ~36 membrane proteins to COPII vesicles involved in the secretory pathway [43,44,45,46]. Although the deletion of Erv14 was shown to cause defects in various cell processes [42,45,46,47], the functions of the protein in plant pathogenic filamentous fungus have not been clearly defined.

In this work, we characterized the integral membrane protein FgErv14 in *F. graminearum*. We show that, as an ER cargo receptor, FgErv14 has important functions in DON production, vegetative growth, asexual and sexual reproduction, and pathogenicity in *F. graminearum*. FgErv14 is mainly located in the ER and participated in both ER-to-Golgi and late Golgi-to-plasma membrane (PM) transport. Moreover, under toxin-inducing conditions, FgErv14 is localized in the toxisome and is required for toxisome generation. Collectively, these results show that the ER cargo receptor FgErv14 is an important regulator of DON production, development, and full virulence in *F. graminearum*.

## 2. Materials and Methods

### 2.1. Fungal Strains and Growth Conditions

The *F. graminearum* gene single deletion mutants were generated from the parental wild-type (WT) PH-1 strain, and all fungal strains used in this study are listed in Supplementary Table S1. For *FgERV14* disruptions, the endogenous copy of *FgERV14* was replaced with a hygromycin resistance (Hyg R) cassette in WT strain PH-1. PCR and Southern blotting were used to identify Δ*Fgerv14* deletion mutants [48]. To generate complemented strains, 927-bp *FgERV14* 5′ promoter region and open reading frames were amplified by PCR from the genome of *F. graminearum*, then, the 3′ end of the PCR product was fused with green fluorescent protein (GFP) and inserted into the pYF11 vector to generate a plasmid expressing FgErv14-GFP. The resulting pYF11-FgErv14-GFP plasmid was transferred into the Δ*Fgerv14* mutant protoplasts using PEG-mediated fungal transformation. The transformants were selected on a G418-resistance medium and identified by PCR and GFP signals [49]. Strains expressing FgRud3-GFP/GFP-FgSnc1-PEM/FgErv14-GFP and FgSed5-mCherry/FgKex2-tdTomato/Tri4-mCherry under their respective native promoters were also obtained by PEG-mediated transformation and selected on a G418-resistance medium and nourseothricin (NAT)-resistance medium, respectively. V8 medium, 5 × yeast extract-glucose (YEG), complete medium (CM), or minimal medium (MM) were used for vegetative growth of fungal strains at 25 °C for 3 days [48]. The conidiation production was determined by culturing each strain in carboxymethyl cellulose (CMC) liquid medium. Perithecium and ascospore generation were induced by carrot agar medium. Trichothecene biosynthesis induction (TBI) medium was used to analyze trichothecene production. To examine mycelial growth under different stress conditions, CM supplemented with 1 M NaCl, 1 M sorbitol, 0.05% sodium dodecyl sulfate (SDS), and 5 mM H_2_O_2_ were used separately [50]. Genomic DNA and RNA were extracted from mycelia harvested from all strains that were cultured in CM for 2 days.

### 2.2. Assessment of Conidia Germination and Perithecial Development

For conidia germination assay, five mycelial plugs (5 mm in diameter) of all strains were taken from the edge using a 50-mm punch and induced in CMC liquid medium at 25 °C for 5 days [51]. Then, the number of conidia was determined by the hemocytometer. The conidia germination was determined by suspending fresh conidia in yeast extract peptone dextrose (YEPD) liquid medium for 4 h, 6 h, and 8 h, and then observing them using the upright microscope (Eclipse Ni-U; Nikon, Tokyo, Japan) [48]. Sexual reproduction was assayed by first inoculating each strain on CA medium at 25 °C for 7 days. After removing the aerial hyphae, each strain was pressed down gently using 1 mL of 2.5% sterile Tween 20 solution [52]. The perithecium formation was determined and photographed after further inducing at 25 °C under black light for about 2–3 weeks.

### 2.3. Plant Infection Assays

Jimai 22 was used in this assay as it was a native susceptible wheat cultivar. Infection of flowering wheat inflorescences was performed as described previously [53]. Three-day-old conidia were collected and spore suspension with a concentration of 4 × 10^5^ spores/mL was prepared. 10 µL of the suspension was injected into the flowering wheat spikelets. To maintain humidity of the wheat heads after injection, the ziplock bags were used after spraying with sterile distilled water. The bags were removed after 2 days, and the wheat heads showing symptoms of wheat head blight disease were collected and photographed two weeks post-inoculation. The disease index was quantified from 40 wheat heads of each strain incubation. For wheat coleoptile infection assay, 2.5 μL of three-day-old conidia suspensions (2 × 10^6^ spores/mL) was used and the samples were photographed 8 days post-inoculation. The lesion length was quantified from 15 plants of each strain incubation.

### 2.4. DON Production and Quantitative Real-Time PCR (qRT-PCR) Analysis

DON biosynthesis was tested by culturing 3 fresh mycelial plugs of each strain on 5 g of wheat grains [54]. The production of DON and ergosterol production were analyzed using the plugs incubated at 25 °C for 20 days [50].

Total RNA extraction was performed as previously described [50]. The *TRI* genes expression levels were detected using qRT-PCR with the appropriate primers and liquid trichothecene biosynthesis induction (TBI) medium induction. The *FgRPS16* gene in *F. graminearum* served as an internal control for normalization of gene expression levels [48], and data of qRT-PCR were calculated using the 2^−ΔΔCT^ method [55].

### 2.5. Microscopic Observations

Each fungal strain expressing fluorescence-tagged proteins from plasmids and conidia stained with calcofluor white (CFW) were examined via fluorescence microscopy using confocal microscopy (LSM 880 NLO system; Zeiss, Oberkochen, Germany) or an upright fluorescence microscope (Eclipse Ni-U; Nikon) [27]. At least 10 different microscopic fields were observed for each sample with one or two channels (Zeiss confocal microscopy: 488 nm for GFP and 561 nm for mCherry/tdTomato. Nikon fluorescence microscopy: UV filter for CFW staining and GFP/RFP filters for fluorescence-tagged proteins). Images were acquired and analyzed using ImageJ and Adobe Photoshop.

### 2.6. Statistical Analyses

The experimental data are displayed as the mean ± standard deviation (SD) from at least three replicates. Duncan’s test and analysis of variance (ANOVA) were performed using GraphPad Prism 8.0 to compare individual samples across different strains or treatments.

## 3. Results

### 3.1. Identification and Knockout of the FgErv14 Protein in F. graminearum

**Figure 1:**
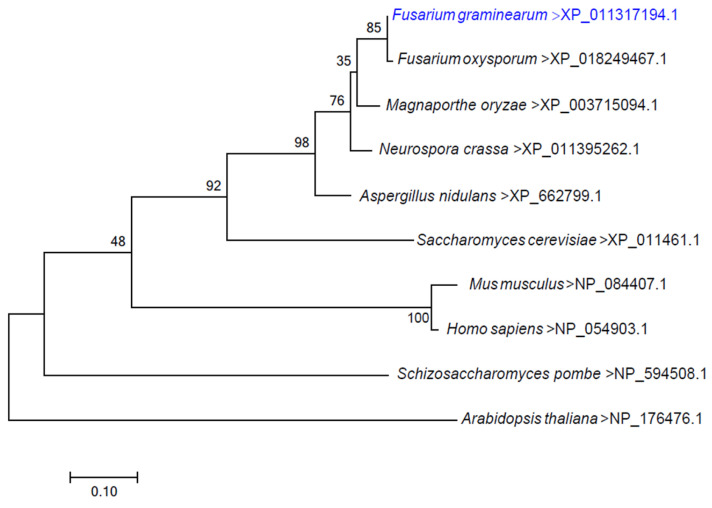
Phylogenetic analysis of *F. graminearum* Erv14 protein and its orthologs in other fungi, including *Fusarium oxysporum* XP_018249467.1, *Magnaporthe oryzae* XP_003715094.1, *Neurospora crassa* XP_011395262.1, *Aspergillus nidulans* XP_662799.1, *Saccharomyces cerevisiae* NP_011461.1, and *Schizosaccharomyces pombe* NP_594508.1. The phylogenetic tree was constructed with mega7.0.

The conserved integral membrane protein FgERV14 (FGSG_01398) of *F. graminearum* was detected using *S. cerevisiae* Erv14 (YGL054C) for a homology search. The *FgERV14* gene is 629 bp in length and encodes a protein of 139 amino acids. From a protein alignment analysis, it was found that FgErv14 exhibited a high degree of identity to other fungal Erv14 proteins of *Fusarium oxysporum* (99.28%), *Magnaporthe oryzae* (92.70%)*, Neurospora crassa* (91.97%), *Aspergillus nidulans* (87.59%), *S. cerevisiae* (62.69%) and *Schizosaccharomyces pombe* (45.54%) (Figure S1A). A phylogenetic analysis showed that Erv14 is widespread in different eukaryotes (Figure 1). Furthermore, a functional domain analysis revealed that Erv14 has three conserved transmembrane domains in fungi, animals, and plants (Figure S1B). The amino acid sequence and domain similarity are more closely related to the FgErv14 protein, which might exhibit a conserved function in *F. graminearum*. To investigate the function of FgErv14, Δ*Fgerv14* mutants were constructed using a homologous recombination strategy (Figure S2A) and were identified by Southern blot analysis (Figure S2B).

### 3.2. Involvement of FgErv14 in Vegetative Growth and Stress Resistance in F. graminearum

**Figure 2:**
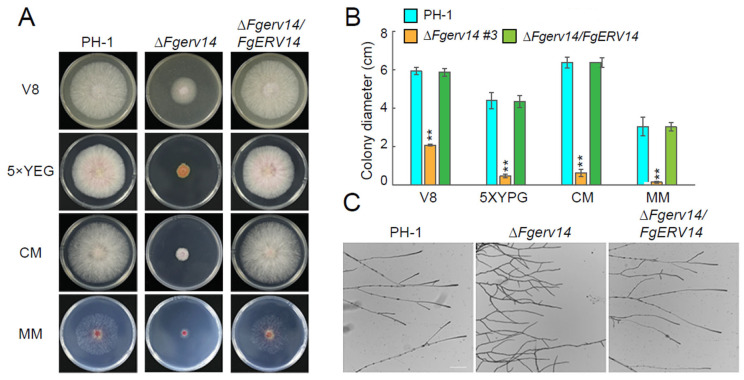
FgErv14 contributes significantly to vegetative growth in *F. graminearum*. (**A**) The Δ*Fgerv14* mutants exhibit a defective colony growth morphology. All strains growth on V8, 5× YEG, CM, and MM medium were tested at 25 °C for 3 days. (**B**) The colony diameters of each strain in (**A**) were quantified. Average values from three experiments are shown. *p* values, **, *p* < 0.01. (**C**) Hyphal growth and the hyperbranching of each strain. Bar, 100 μm.

**Figure 3:**
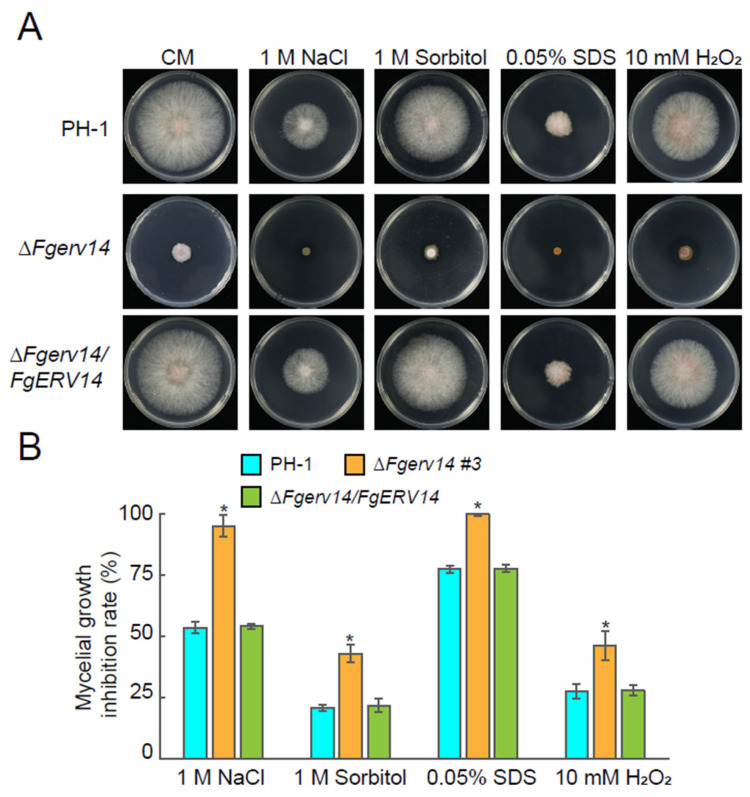
FgErv14 is involved in vegetative growth under different stress conditions. (**A**) Colonies of each strain were grown on CM medium containing various stress-inducing agents at the indicated concentrations. All strains were incubated at 25 °C for 3 days. (**B**) The growth inhibition rate of each strain under different stress conditions in (**A**) were analyzed statistically. The inhibition rate was calculated as: Inhibition rate (%) = (the diameter of untreated strain − the diameter of treated strain)/(the diameter of untreated strain ×  100%). Error bars mean SD. *p* values, *, *p* < 0.05.

In *S. cerevisiae*, the absence of Erv14, a non-essential protein, does not affect vegetative growth [38,56]. The role of *F. graminearum* FgErv14 in vegetative growth was investigated by comparing WT PH-1, Δ*Fgerv14* mutants (#3 and #4), and a Δ*Fgerv14*/*FgERV14* complemented strain cultured on V8 juice medium. The Δ*Fgerv14* mutant strains exhibited a phenotype of defective vegetative growth compared to that in PH-1 and the Δ*Fgerv14*/*FgERV14* strain (Figure 2A,B). To further characterize whether the vegetative growth defects were medium dependent, each strain was grown on YEG, CM, and MM plates. Under all three conditions, the vegetative growth in Δ*Fgerv14* mutants was also defective compared to that in PH-1 and the Δ*Fgerv14*/*FgERV14* strain, consistent with the growth on the V8 medium. An examination of the responses of the mutants to osmotic stress (NaCl, sorbitol), cell wall disruption (SDS), and oxidative stress (H_2_O_2_) revealed a significant inhibition of growth in the Δ*Fgerv14* strain compared to WT and Δ*Fgerv14*/*FgERV14* strains (Figure 3), suggesting that FgErv14 is important for osmoregulation and stress tolerance. To extend our detection, the hyphae of each strain were examined, and the Δ*Fgerv14* mutant showed extensive branching hyphae in contrast to the WT and complementary strains (Figure 2C). These results imply that FgErv14 regulates mycelial growth positively in *F. graminearum*.

### 3.3. FgErv14 Controls F. graminearum Asexual and Sexual Reproduction

**Figure 4:**
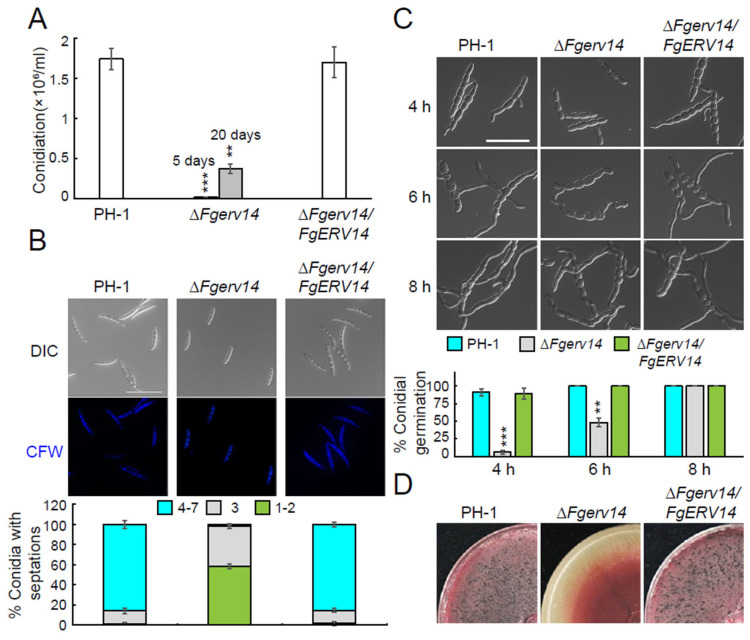
The asexual and sexual reproduction were defective in Δ*Fgerv14* mutant. (**A**) Conidia production of each strain. Conidia harvested from CMC medium after incubation for quantitative analysis. Error bars mean SD. *p* values, **, *p* < 0.01, ***, *p* < 0.001. (**B**) Conidia of specified strains were stained with CFW and the spore septa were observed via fluorescence microscopy. Bar, 50 μm. The percentages of conidia producing different numbers (<3, =3, and 4–7) of septa were recorded. Error bars represent SDs. (**C**) Conidia of specified strains were cultured in liquid YEPD medium, and the germination rate after 4, 6, and 10 h were observed via microscopy. Bar, 50 μm. Error bars mean SDs. *p* values, **, *p* < 0.01; ***, *p* < 0.001. (**D**) Each strain was inoculated on the CA medium for 7 days to detect the perithecium generation.

The role of FgErv14 in asexual development was explored by examining whether FgErv14 is required for conidial production. As shown in Figure 4A, after 5 days of culture on CMC medium, the conidial production of Δ*Fgerv14* mutant was significantly lower than that of PH-1 and the Δ*Fgerv14*/*FgERV14* strains. After 20 days, the conidial production of Δ*Fgerv14* mutant was still much lower than that of PH-1 counted on the 5th day (Figure 4A), indicating that FgErv14 plays an essential role in conidial production. To further investigate conidial morphology, CFW staining was used to observe the conidia of each strain. The data showed that 39.9% and 58.4% of conidia in the Δ*fgerv14* mutant had 3 and 1–2 septa, respectively (Figure 4B). By contrast, the conidia production in WT PH-1 and the Δ*Fgerv14*/*FgERV14* strains were largely normal, with 4–7 septa produced by 85.9% and 85.2% of the conidia, respectively (Figure 4B). Upon further analysis, the Δ*Fgerv14* mutant was shown to exhibit delayed germ tube development compared to WT and Δ*Fgerv14*/*FgERV14* strains (Figure 4C), suggesting that FgErv14 is involved in germ tube germination and hence, in the conidial production in *F. graminearum*.

The sexual reproduction of *F. graminearum* is the main step in the FHB disease cycle. Therefore, we examined perithecium generation in the Δ*Fgerv14* mutant. PH-1 and Δ*Fgerv14*/*FgERV14* strain growth on carrot agar medium for the sexual reproduction assay produced abundant perithecia at 2 weeks post-fertilization, whereas no perithecia were produced by the Δ*Fgerv14* mutant (Figure 4D), indicating that FgErv14 plays a crucial role in the sexual reproduction of *F. graminearum*.

### 3.4. FgErv14 Is Important for F. graminearum Virulence

**Figure 5:**
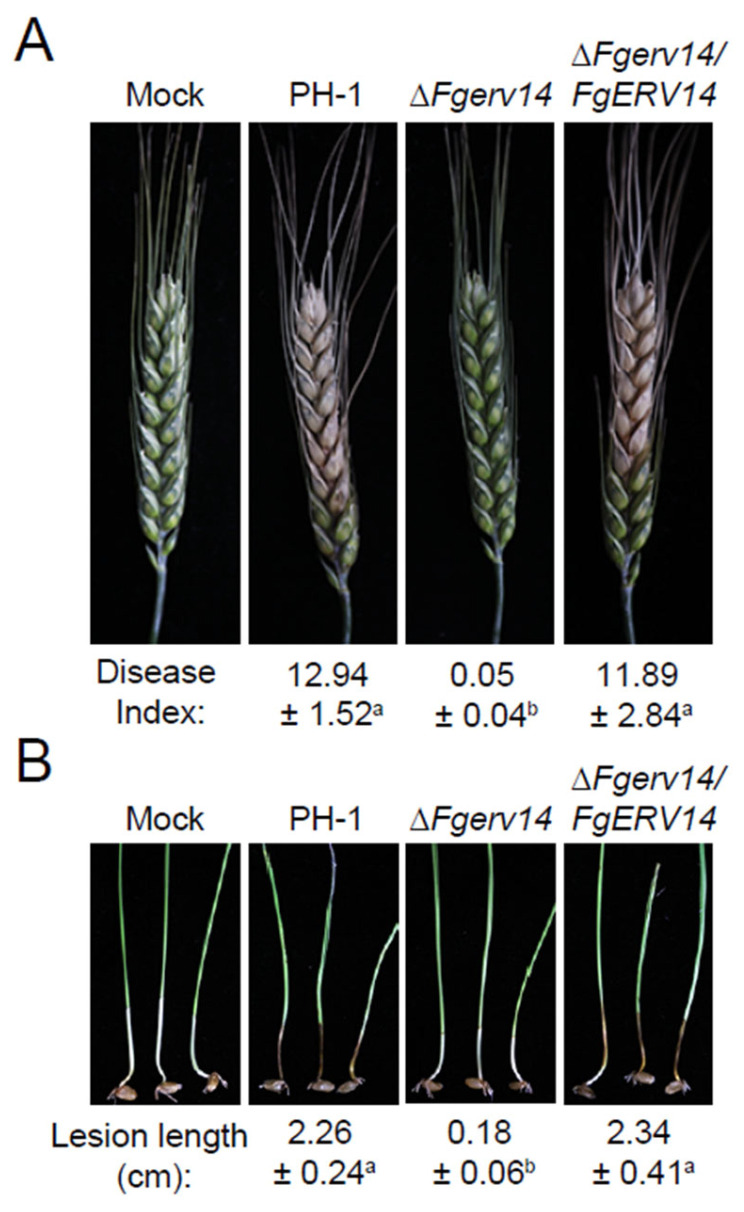
FgErv14 is necessary for full virulence of *F. graminearum*. (**A**) Conidial suspensions of each strain were used and the wheat heads were collected and photographed two weeks post inoculation. Differences in mean (± SD) values are indicated by different lowercase letters at *p* < 0.01 level. (**B**) Wheat coleoptiles were infected with conidial suspensions and investigated after 10 days. The same letters represent no significant differences at level of *p* < 0.01. Error bars mean SDs.

To investigate the effects of FgErv14 in *F. graminearum* virulence, flowering wheat spikelets were inoculated by spore suspension with a concentration of 4 × 10^5^ conidia/mL to test the virulence of each strain. At 2 weeks after inoculation, it was found that WT and the Δ*Fgerv14*/*FgERV14* strain caused obvious symptoms of head blight, with an average disease index (the number of spikelets showing disease symptoms per head) of 12.94 and 11.89, respectively, indicating full virulence (Figure 5A). By contrast, the Δ*Fgerv14* mutant failed to spread around and no typical symptoms were observed in wheat flowering heads, with an average disease index of 0.05, indicating significantly attenuated virulence (Figure 5A). Similar results were also obtained in the wheat coleoptiles assays. In contrast to the obvious lesion caused by PH-1 and the Δ*Fgerv14*/*FgERV14* strain, the Δ*Fgerv14* mutant induced limited lesion development only around the inoculation sites (Figure 5B). These results re-establish that FgErv14 is involved in *F. graminearum* full virulence during plant infection.

### 3.5. FgErv14 Is Necessary for the Production of DON in F. graminearum

**Figure 6:**
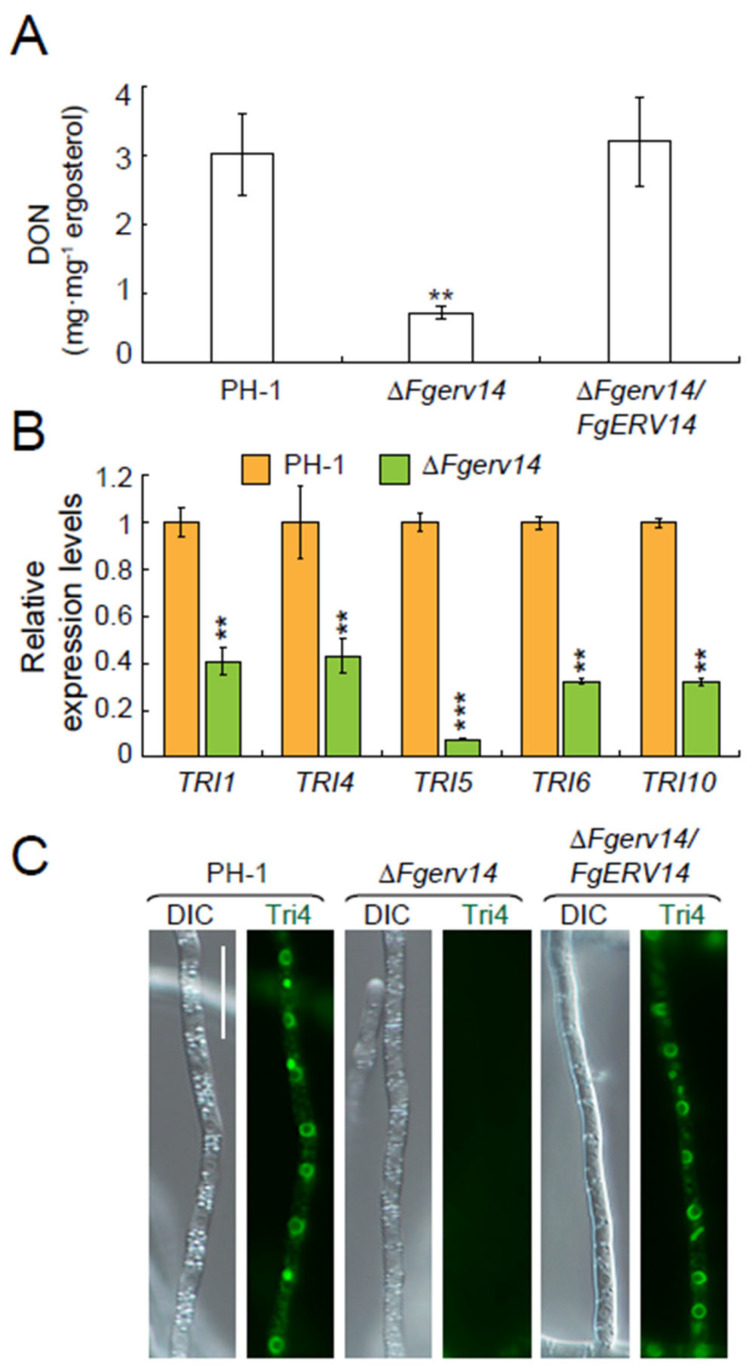
FgErv14 is critical for DON production. (**A**) DON production level was determined in wheat grains infected with each strain. Error bars mean SDs. **, *p* < 0.01. (**B**) Relative expression folds of key genes in the DON biosynthetic pathway including *TRI1*, *TRI4*, *TRI5*, *TRI6*, and *TRI10* were monitored by qRT-PCR in specified strains. The *FgRPS16* gene served as the internal control. Error bars mean SDs. *p* values, **, *p* < 0.01; ***, *p* < 0.001. (**C**) Strains of PH-1 and the Δ*Fgerv14* expressing Tri4-GFP were observed under the induction of TBI medium. Fresh mycelia were observed using fluorescence microscopy. Bars, 10 μm.

A secondary metabolite mycotoxin DON is essential for *F. graminearum* virulence [11,57]. Therefore, we assessed DON biosynthesis in PH-1, the Δ*Fgerv14* mutant and Δ*Fgerv14*/*FgERV14* strains. As shown in Figure 6A, DON production by the Δ*Fgerv14* mutant was 25% of that by WT PH-1 or the Δ*Fgerv14*/*FgERV14* strain, with a DON level of only 0.71 mg ergosterol·mg^−1^. The expression of *some TRI* genes responsible for the regulation of DON production were examined. Consistent with the reduction in the production of DON in the Δ*Fgerv14* mutant, the qRT-PCR results showed that the expression levels of *TRI1*, *TRI4*, *TRI5*, *TRI6*, and *TRI10* in the Δ*Fgerv14* mutant were significantly lower than that of PH-1 (Figure 6B), indicating that FgErv14 is essential for modulating the expression of *TRI* genes. Previous studies suggested that DON is synthesized in the toxisome localized in the ER [18,58]. Therefore, further monitoring of the toxisome formation was carried out by visualizing the localization of Tri4-GFP in each strain. As shown in Figure 6C, the Tri4-GFP signals from both PH-1 and Δ*Fgerv14*/*FgERV14* strains were clustered in a crescent-shaped structure in hyphae, whereas no signal was observed in the Δ*Fgerv14* mutant. Together, our results indicate that the defection of DON production in Δ*Fgerv14* mutant was due to the essential role of FgErv14 in the formation of toxisome in *F. graminearum*.

### 3.6. FgErv14 Is Mainly Localized in the ER and Toxisome in F. graminearum

**Figure 7:**
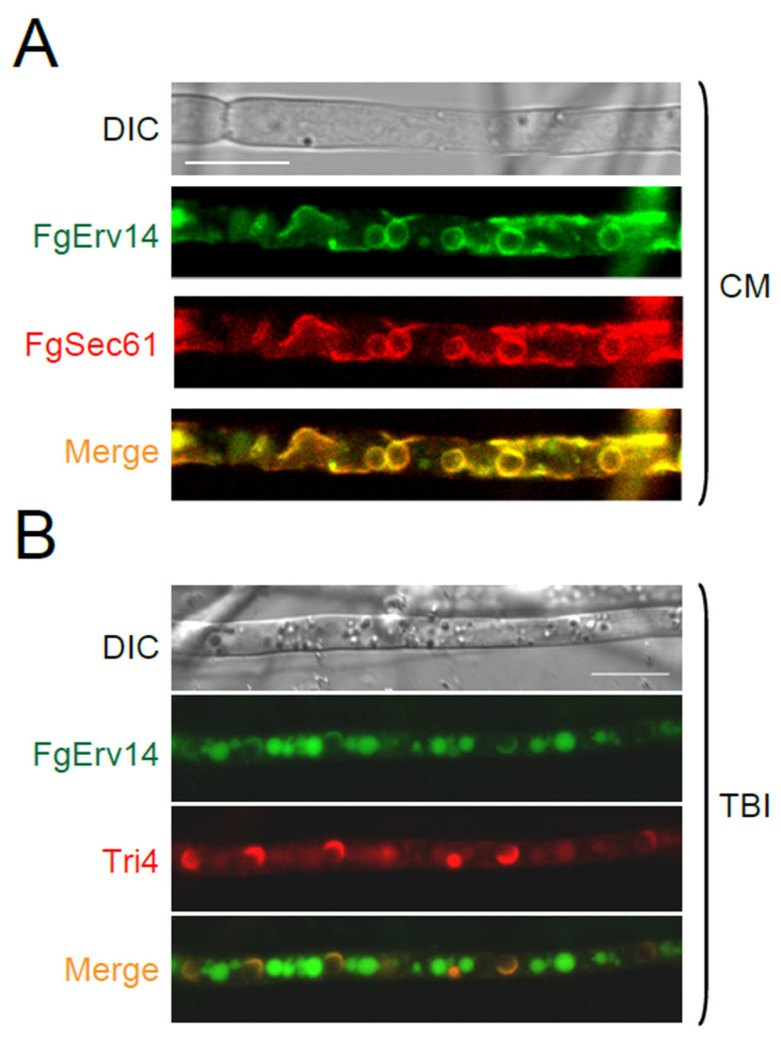
FgErv14 localizes to the ER and toxisome. (**A**) FgErv14-GFP and FgSec61-mCherry were expressed in WT PH-1 cultured in CM. (**B**) FgErv14-GFP and FgTri4-mCherry were expressed in WT PH-1 cultured with TBI medium. The hyphae from (**A**,**B**) were examined via live cell fluorescence microscopy. Bars, 10 μm.

The function of FgErv14 was further investigated by constructing the FgErv14-GFP fusion protein and then examining its cellular localization in *F. graminearum*. It has been reported that the *S. cerevisiae* Erv14 is mainly present on the ER [56]. Accordingly, a PH-1 strain co-expressed FgErv14-GFP with the ER marker FgSec61-tdTomato was obtained. The results showed that on CM, FgErv14-GFP colocalized with FgSec61-tdTomato (Figure 7A), indicating that the subcellular localization of FgErv14 is in the ER, consistent with Erv14 in *S. cerevisiae*. Furthermore, as FgErv14 is involved in toxisome formation (Figure 6C), strains that co-expressed FgErv14-GFP with toxisome marker Tri4-mCherry were constructed. Under toxin-inducing conditions, we found that FgErv14-GFP either accumulated in a crescent-shaped structure or dispersed in a circle within hyphae (Figure 7B), and the FgErv14-GFP signals colocalized with Tri4-mCherry (Figure 7B), indicating the presence of FgErv14 in the toxisome. These results indicate that toxin-inducing conditions is a key factor in the localization of FgErv14 to toxisomes.

### 3.7. FgErv14 Is Required for FgRud3 and FgSnc1-PEM Localization in F. graminearum

**Figure 8:**
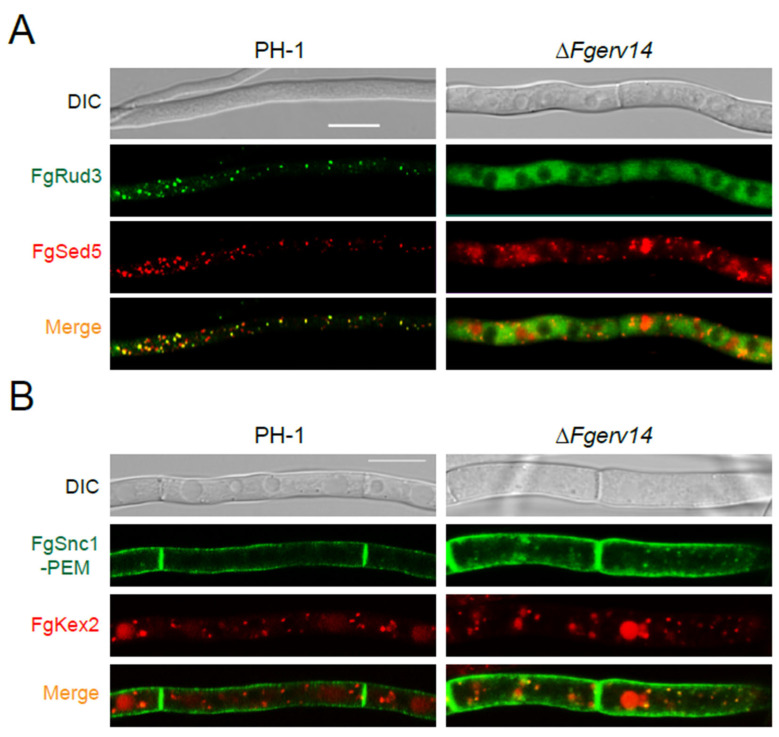
FgErv14 is involved in the localization of FgRud3 and the transport from Golgi-to-PM in *F. graminearum*. (**A**) Expression of chromosomally tagged FgRud3-GFP and mCherry-FgSed5 in PH-1 and the Δ*Fgerv14* mutant were imaged using confocal microscopy. (**B**) FgErv14 is involved in the transport between late Golgi and PM. GFP-FgSnc1-PEM and FgKex2-tdTomato were co-expressed in specified strains. Bars, 10 μm.

In yeast, Erv14 is a component of COPII vesicles that regulate ER-to-Golgi transport [38]. During this process, Erv14, as an ER cargo receptor, regulates cargo protein loading during ER exit [56]. FgRud3 is an early-Golgi localization protein required for toxisome generation and virulence in *F. graminearum* [27]. Whether FgErv14 affects the localization of FgRud3 was determined by monitoring FgRud3-GFP signals in PH-1 and the Δ*Fgerv14* mutant strains. In the former, FgRud3-GFP colocalized with mCherry-FgSed5 (Figure 8A), the early-Golgi marker, whereas in the mutant lacking *FgERV14*, it was instead diffusely distributed throughout the cytosol (Figure 8A), implying that the normal localization of FgRud3 depends on FgErv14. We then used late secretory pathway markers (GFP-FgSnc1-PEM: mutant V42A and M45A) to investigate whether FgErv14 is involved in the late secretory pathway, which mediates trafficking from late Golgi to PM [27]. In WT PH-1 hyphae, GFP-FgSnc1-PEM accumulated on the PM, whereas in hyphae of the Δ*Fgerv14* mutant, although the signal was mostly localized to the PM, multiple green dots were also detected in the cytoplasm (Figure 8B) colocalized with the late Golgi marker FgKex2-tdTomato, indicating that GFP-FgSnc1-PEM was partly trapped in the late Golgi. These results suggest that there are defects in the Golgi-to-PM transport, but it was not completely blocked in the Δ*Fgerv14* mutant.

## 4. Discussion

Vesicular transport from the ER to the Golgi in the early secretory pathway is necessary for growth and development in eukaryotes, including phytopathogenic fungi [27,34,36,37,59]. Erv14, an ER cargo receptor, circulates between the ER and Golgi apparatus and participates in the direct selection of COPII cargo proteins [38,43,45,46,47]. Although Erv14 and its related proteins have been described in yeast and mammalian cells, their function in phytopathogenic fungi is still unclear. In this study, we identified the corresponding ER cargo receptor FgErv14 in *F. graminearum*. Our results demonstrate a role of FgErv14 in vesicle transport and thus, involvement in the control of the DON production, vegetative growth, asexual and sexual reproduction, and pathogenesis of *F. graminearum*.

In *S. cerevisiae*, *ERV14* is a non-essential gene, and its absence does not affect vegetative growth [38,56]. However, in contrast to the phenotype of the *erv14*Δ yeast mutant, our results showed that the vegetative growth was reduced significantly in the Δ*Fgerv14* mutant compared to WT (Figure 2A,B). Secretory trafficking is very important for the vegetative growth of filamentous fungi, and it was reported that a blockade of early secretory transport causes defects in vegetative growth in *A. nidulans* [60,61]. Therefore, it suggests that the growth defect of ΔFgerv14 mutant is related to FgErv14-mediated secretory transport. Moreover, the deletion of FgErv14 mutant was more hypersensitive to the osmotic, cell wall, and oxidative stresses (Figure 3), which is consistent with Erv14 deletion mutant results in greater sensitivity to osmotic stress in *S. cerevisiae* [46,47]. Thus, like *S. cerevisiae* Erv14, FgErv14 may affect partial Na^+^, K^+^/H^+^ exchanger/antiporter (Nha1 [56]) localization by mediating vesicular trafficking, which leads to a more sensitive phenotype to osmotic and oxidative in Δ*Fgerv14* mutant. As expected, the Δ*Fgerv14* mutant produced significantly fewer conidia than WT, and the mutant also produced a higher proportion of morphological and germination defective conidia (Figure 4B,C). Although this phenotype is consistent with those resulting from the partial absence of other COPII components and regulators, such as FgSec22 and FgRab1 [34,36], the defect level in the Δ*Fgerv14* mutant was higher.

During the FHB disease cycle, the sexual reproduction of *F. graminearum* is the main source of infection of plants in nature [62,63]. Previous studies have shown that the COPII components FgSec22 and FgRab1 are not necessary for sexual reproduction in *F. graminearum* [34,36]. By contrast, we found that FgErv14 regulates perithecium formation and is required for sexual reproduction (Figure 4D), suggesting that FgErv14 plays an important role in *F. graminearum* infection of plants. However, as the roles of FgSec22 and FgRab1 in regulating ER-to-Golgi transport in *F. graminearum* have not been determined, their contributions to early secretory transport during sexual reproduction is unclear. In yeast, Erv14 regulates prospore membrane formation and affects sexual reproduction through an as-yet unknown mechanism. The mechanism of FgErv14 regulating *F. graminearum* sexual reproduction needs to be further studied.

DON is the most toxic secondary metabolite produced by *F. graminearum*, and its production is affected by various physiological processes [16,64]. The protein encoded by the *TRI* genes are distributed in different steps to catalyze DON production [16]. During DON biosynthesis, its derivatives will also be produced [65]. In DON quantification, LC-MS technology is used to distinguish different derivatives. In the Δ*Fgerv14* mutant, the expression of *TRI* gene was decreased, and the DON production was also greatly reduced in LC-MS determination. This result is consistent with many vesicle trafficking regulators, like Rab GTPases [30], FgVam7 [25], and FgLsb6 [66]. Together, FgErv14 plays a crucial role in DON biosynthesis.

The ER cargo receptor Erv14 is highly conserved in eukaryotes, but it does not mediate the transport of all newly synthesized proteins. The golgin protein Rud3, the general amino acid permease Gap1, and the Na^+^/H^+^ antiporter are proteins transported by yeast Erv14 [45,56,67]. In this study, our results showed that FgErv14 was responsible for the localization of FgRud3 in the early-Golgi of *F. graminearum* (Figure 8A), demonstrating evidence of its function as an ER cargo receptor that mediates the COPII vesicle-associated trafficking from the ER to Golgi. Our previous studies showed that FgRud3 regulates the pathogenicity of *F. graminearum* and affects toxisome formation in the mycelium, consistent with the phenotype of the Δ*Fgerv14* mutant. Thus, at least some of the COPII cargo proteins selected by FgErv14 likely regulate pathogenicity and toxisome generation in *F. graminearum*, pointing to the regulatory role of transport of COPII cargo proteins. Under toxin-inducing conditions, FgErv14 is partially localized in the toxisome, suggesting its involvement in toxisome generation. We also observed that GFP-FgSnc1-PEM was partially trapped in the late Golgi apparatus in the Δ*Fgerv14* mutant, whereas most of the GFP-FgSnc1-PEM is normally transported to the PM (Figure 8B). According to these results, FgErv14 also affects late secretory transport from the late Golgi to the PM, but its absence does not completely block this pathway. In *A. nidulans*, the absence of SarA(Sar1) or RabO(Rab1) blocks ER-to-Golgi transport completely, resulting in the reabsorption of early Golgi cisternae into the ER, whereas late Golgi-associated proteins were dispersed in the cytoplasm [60,61,68]. Our results show that FgSed5 formed punctate structures and did not exhibit an ER localization phenotype in the Δ*Fgerv14* mutant, indicating that FgErv14 does not regulate the localization of FgSed5. This result is consistent with the fact that FgErv14 does not mediate all COPII anterograde transport. Given that FgErv14 has only an apparent ER localization (Figure 7A), we speculate that partial regulators of late secretory transport are abnormally localized in the Δ*Fgerv14* mutant, resulting in defective late secretory transport. As secretory transport contributes significantly to the DON production, virulence, growth, and development of *F. graminearum* [28,69], FgErv14 may affect pathogenicity by regulating this process. Thus, these results support that FgErv14 regulates the transport of ER-to-Golgi and that this process affects Golgi-to-PM transport in *F. graminearum*.

## 5. Conclusions

In summary, our study demonstrated the involvement of the ER cargo receptor FgErv14 in vegetative growth, DON production, asexual and sexual reproduction, and pathogenicity in *F. graminearum*. FgErv14 was observed in the toxisome, where it plays an important role in toxisome generation. We also found that FgErv14 participates in ER-to-Golgi transport and thus, in the regulation of *F. graminearum* virulence.

## Data Availability

Data is contained within the article.

## Supplementary Materials

The following supporting information can be downloaded at: https://www.mdpi.com/article/10.3390/life12060799/s1, reference [70] is cited in the Supplementary Materials. Figure S1: Amino-acid sequence and domain alignment of Erv14; Figure S2: Generation and verification of *FgERV14*-deletion mutants; Table S1: *F. graminearum* strains used in this study.
