# Supplementary material for: The Endoplasmic Reticulum Cargo Receptor FgErv14 Regulates DON Production, Growth and Virulence in Fusarium graminearum
Source: Life (Basel). 2022 May 27;12(6):799. doi: 10.3390/life12060799 (PMC9224835; doi:10.3390/life12060799)
Supplement: Supplementary file 1 [file life-12-00799-s001.zip › life-1702625-supplementary.pdf]

Supplementary Information

The endoplasmic reticulum cargo receptor FgErv14 regulates DON production, growth and virulence in *Fusarium graminearum*

Sun *et al*.

Table S1. *F. graminearum* strains used in this study

Strain	Genotype	Source	Figures
FDH69	PH-1 (Wide Type)	[70]	Fig.2A-C Fig.3A-D Fig.5A-B Fig.6A-B Fig.S2B Fig.3A-B
FHD655	PH-1, Δ*Fgerv14::HYG* #3	This study	Fig.2A-C Fig.4A-D Fig.5A-B Fig.6A-B Fig.S2B Fig.3A-B
FHD657	PH-1, Δ*Fgerv14::HYG* #4	This study	Fig.2A-C Fig.S2B
FHD500	FHD657, *FgERV14-GFP::KAN*	This study	Fig.2A-C Fig.4A-D Fig.5A-B Fig.6A-B Fig.S2B Fig.3A-B
FHD581	PH-1, *TRI4-GFP:: KAN*	This study	Fig.6C
FHD818	FHD657, *TRI4-GFP:: KAN*	This study	Fig.6C
FHD635	PH-1, *FgERV14-GFP::KAN*, *FgSEC61-mCherry::NAT*	This study	Fig.7A
FHD651	PH-1, *FgERV14-GFP::KAN*, *TRI4-mCherry::NAT*	This study	Fig.7B
FHD551	PH-1, *FgRUD3-GFP::KAN*, *FgSED5-mCherry::NAT*	[2]	Fig.8A
FHD808	FHD657, *FgRUD3-GFP::KAN*, *FgSED5-mCherry::NAT*	This study	Fig.8A
FHD569	PH-1, *mCherry-FgKEX2::KAN*, *GFP-FgSNC1-PEM::NAT*	This study	Fig.8B
FHD960	FHD657, *mCherry-FgKEX2::KAN*, *GFP-FgSNC1-PEM::NAT*	This study	Fig.8B

Supplementary Figures

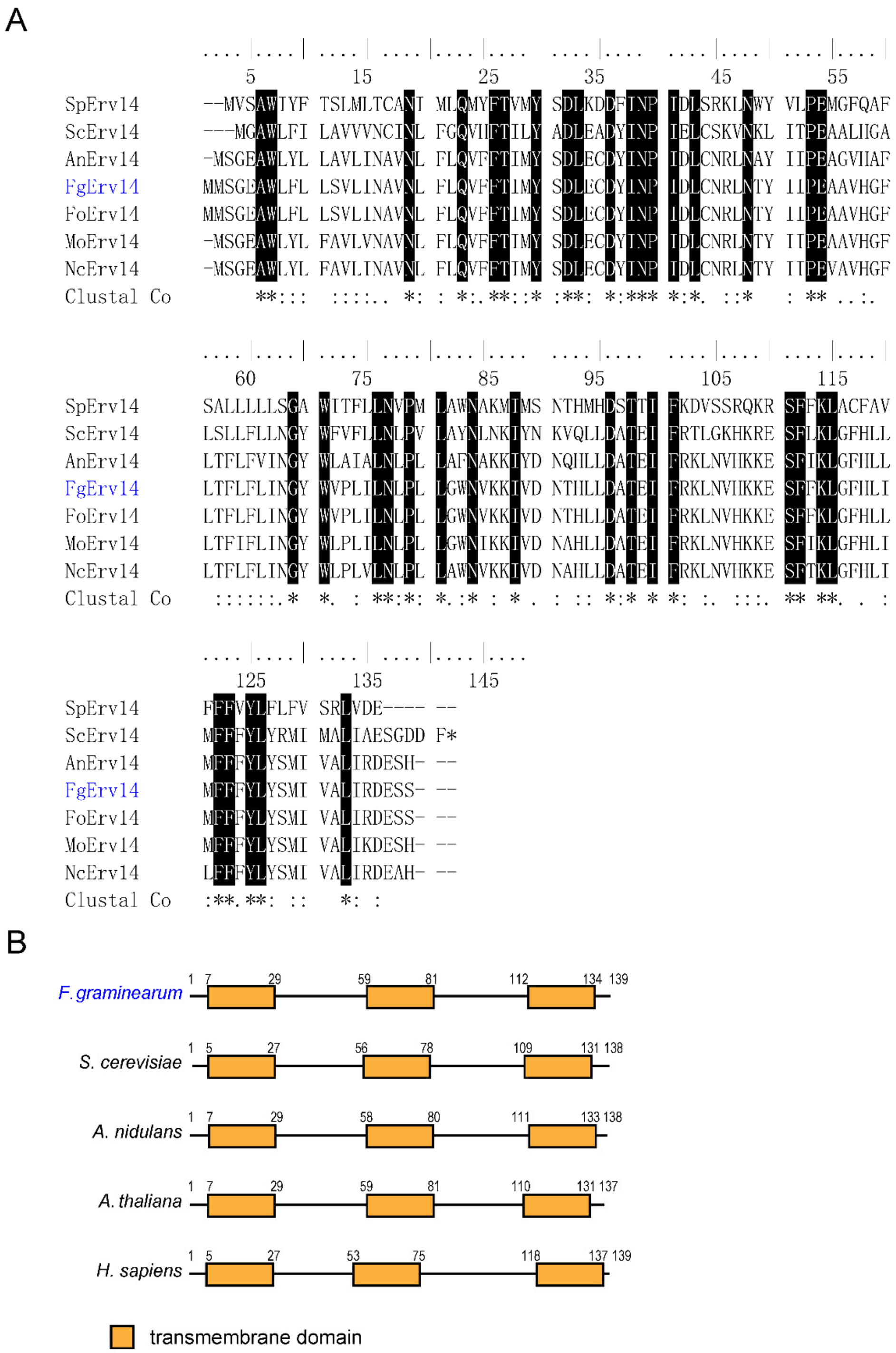

Figure S1. Amino-acid sequence and domain alignment of Erv14. (A) Identical segments are indicated by a black background. The compared sequences were from *F. graminearum*, *F. oxysporum*, *M. oryzae*, *N. crassa*, *A. nidulans*, *S. cerevisiae*, and *S. pombe*. (B) Conserved domains of Erv14 proteins are found in *F. graminearum*, *S. cerevisiae*, *A. nidulans*, *A. thaliana* and *H.sapiens*.

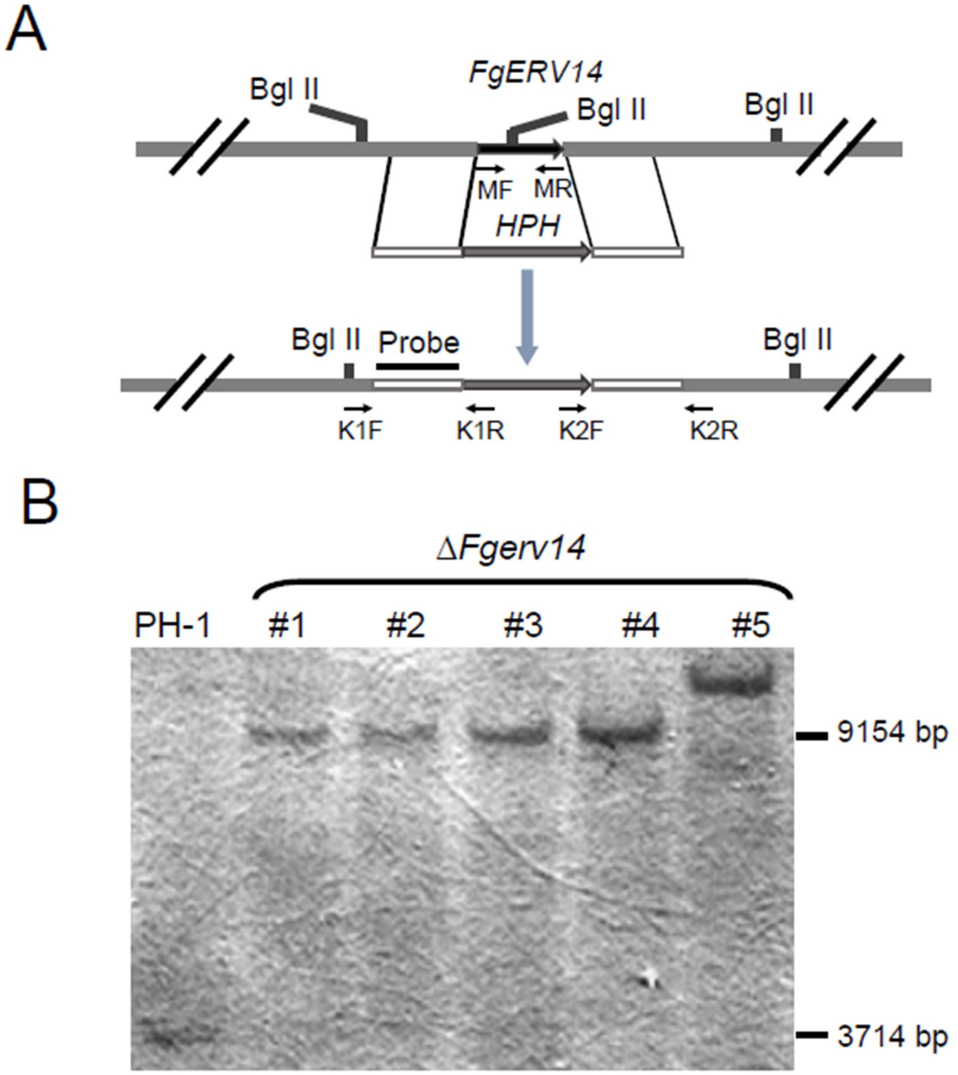

Figure S2. Generation and verification of *FgERV14*-deletion mutants. (A) Strategy for achieving *FgERV14* deletion in *F. graminearum*. The *FgERV14* gene was replaced by a cassette containing a *HPH* gene. (B) Southern blot analysis of WT PH-1 and ΔFgerv14 mutants (#1–5). Genomic DNA was digested with *Bgl*II.
